# Supplementary material for: Dangerous Stops: Nonsense Mutations Can Dramatically Increase Frequency of Prion Conversion
Source: Int J Mol Sci. 2021 Feb 3;22(4):1542. doi: 10.3390/ijms22041542 (PMC7913716; doi:10.3390/ijms22041542)
Supplement: Supplementary file 1 [file ijms-22-01542-s001.zip › ijms-1080443-supplementary final.docx]

Supplementary materials


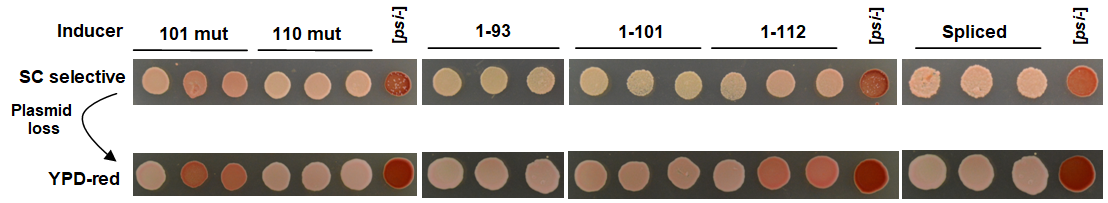


**Figure 1.** [*PSI*^+^] isolates induced by the indicated Sup35 inducer constructs were spotted to SC media selective for the plasmid marker with low adenine content. Then, the inducer plasmid was lost by growth on non-selective YPD medium. The resulting cells were spotted to YPD-red medium. The [*PSI*^+^] prion was kept in all cases, as evident from the pink color of the spots.


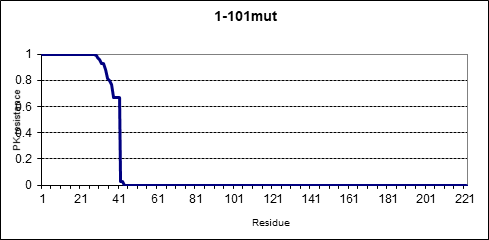

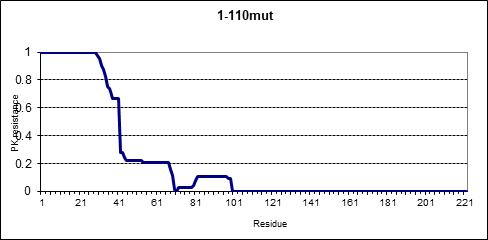


**Figure 2.** Proteinase K resistance profiles of the Sup35NM region from two [*PSI*^+^] prions obtained by expression of the indicated Sup35 nonsense mutant. The PK resistance index was calculated for every residue of Sup35NM as a sum of mass spectral peak areas of resistant peptides which include this residue [15].
